# Exercise-Training in Young *Drosophila melanogaster* Reduces Age-Related Decline in Mobility and Cardiac Performance

**DOI:** 10.1371/journal.pone.0005886

**Published:** 2009-06-11

**Authors:** Nicole Piazza, Babina Gosangi, Shawn Devilla, Robert Arking, Robert Wessells

**Affiliations:** 1 Department of Internal Medicine, Institute of Gerontology, University of Michigan Medical School, Ann Arbor, Michigan, United States of America; 2 Department of Biology, Wayne State University, Detroit, Michigan, United States of America; Roswell Park Cancer Institute, United States of America

## Abstract

Declining mobility is a major concern, as well as a major source of health care costs, among the elderly population. Lack of mobility is a primary cause of entry into managed care facilities, and a contributing factor to the frequency of damaging falls. Exercise-based therapies have shown great promise in sustaining mobility in elderly patients, as well as in rodent models. However, the genetic basis of the changing physiological responses to exercise during aging is not well understood. Here, we describe the first exercise-training paradigm in an invertebrate genetic model system. Flies are exercised by a mechanized platform, known as the Power Tower, that rapidly, repeatedly, induces their innate instinct for negative geotaxis. When young flies are subjected to a carefully controlled, ramped paradigm of exercise-training, they display significant reduction in age-related decline in mobility and cardiac performance. Fly lines with improved mitochondrial efficiency display some of the phenotypes observed in wild-type exercised flies. The exercise response in flies is influenced by the amount of protein and lipid, but not carbohydrate, in the diet. The development of an exercise-training model in *Drosophila melanogaster* opens the way to direct testing of single-gene based genetic therapies for improved mobility in aged animals, as well as unbiased genetic screens for loci involved in the changing response to exercise during aging.

## Introduction

The study of functional senescence is of great importance to the design of interventions to extend quality of life in the elderly population. The most common complaint among those of advanced age is declining mobility [1], [2]. Loss of mobility creates substantial direct and indirect health-care costs. It is frequently cited as the deciding factor in patients being forced to enter managed-care facilities, and is also an important indirect cause in many debilitating injuries due to falls [3]–[6]. Indeed, an index of mobility has been proposed as an effective predictor of the degree of self-care ability an individual elder will retain [7], [8].

An intervention that has demonstrated great promise in maintaining functional mobility in humans and in rodent models is exercise-training. Exercise-training causes profound changes in skeletal muscle capability [9] and cardiac performance [10], [11]. In both humans and rodent models, exercise-training reduces the incidence of several age-related diseases, including cardiovascular disease [12] and Type II Diabetes [13]. Furthermore, non-pathological age-related declines in both cardiovascular function and in mobility are slower in rodents or humans that undergo a regular exercise program [9], [10], [12], [13]. However, the genetic mechanisms by which exercise affects the aging process are not well understood.

In vertebrates, exercise-training has been shown to upregulate mitochondrial biogenesis through an increase in the level of *PGC1α* and to alter energy metabolism, at least in part through regulation of *AMPK* (AMP-activated protein kinase) [14]–[17]. In part because the sirtuin family of histone deacetylases also regulates an overlapping subset of metabolic genes [18], [19], and since the prototypical sirtuin *sir2* has been proposed to mediate lifespan extension by dietary restriction [20], [21], it has been suggested that endurance exercise and dietary restriction work through common mechanisms. In support of this idea, both dietary restriction and endurance exercise improve mitochondrial efficiency [22]–[27], increase the levels of ROS-scavenging enzymes and other stress-response genes [28]–[31] and reduce accumulation of oxidative damage [32]–[34].

On the other hand, experiments in invertebrates have shown an increase in oxidative stress, with a concomitant decrease in lifespan, in animals that undergo a lifelong increase in physical activity [35], [36]. Furthermore, dietary restricted animals do not show increases in mobility or late-life functionality that correlate well with those seen in exercise-trained vertebrates [37]. Most importantly, although lifelong exercise can extend mean lifespan in vertebrate models [38]–[40], it does not appear to extend maximal lifespan, as dietary restriction does in multiple species [41]. Taken together, these observations suggest that, along with some commonalities, significant mechanistic differences also exist between exercise-training and dietary restriction.

In order to facilitate the identification and study of genetic factors that specifically mediate the effects of exercise-training on aging physiology, we have developed a novel exercise-training paradigm in *Drosophila melanogaster*. This training and assay system is suitable for both large-scale invertebrate genetics and for longitudinal studies to follow individual changes in physiology during aging.

Here, we describe changes to activity, mobility and cardiac performance as a result of exercise-training in *Drosophila*. Age-related declines to both mobility and cardiac stress resistance are substantially reduced in exercise-trained flies as compared to genetically identical, unexercised controls. We provide evidence that these changes derive, at least in part, from changes in mitochondrial efficiency, and act through mechanisms partially separable from dietary restriction.

## Methods

### Fly Stocks, Diet and Husbandry

*Y^1^w^1^*, *y^1^w^67c23^* and *Oregon R* were obtained from the Bloomington Stock Center. La and Ra lines were obtained from Robert Arking. Standard diet utilized contains 10% yeast, 10% sucrose and 2.5% agar. Modified diets altered the percentage of either yeast or sucrose without altering agar. During the experimental timecourse, flies are housed in a 25°C incubator with 50% humidity and a 12-hour light/dark cycle.

### Negative Geotaxis Assay

100 flies of each experimental group and control group are reserved for longitudinal climbing assays. Pictures are taken everyday prior to being placed on the machine to avoid effects of fatigue or stress from the machine itself. Five to six empty vials are used, with lines forming four equally spaced quadrants. Flies are allowed to equilibrate to the vials in the 25°C room immediately before each training session for ten minutes before assessing negative geotaxis.

Briefly, with a light box behind the vials, tap the rack down four times and on the fourth time, utilize a timed digital camera to snap a picture after four seconds. The extent of climbing can be analyzed visually or by imaging software. Four pictures of each group are taken and averaged to arrive at a fixed score for each vial. Placing labels directly within the picture itself helps avoid confusion later when analyzing pictures. After the flies are back in their normal vials, spray the assay vials with Antistatic spray, wipe with a Kimwipe, and let dry for 24 hours.

Once photographs are collected, the number of flies in each of four equally-spaced quadrants per vial is charted. Flies are assigned individual scores based on which quadrant they reach within the allotted four seconds. Flies that reach the fourth and highest quadrant are given a score of four, flies in the third quadrant are given three points, flies in the second are given two points, flies in the first one point, while flies that fail to leave the bottom of the vial are scored as zero. The total for all the flies in a vial is tallied, then divided by the number of flies in the vial to generate the “Climbing Index” for that trial. Each vial is subjected to four trials, then the indexes from the four trials are averaged. Finally, the climbing index for each vial on the first day of trials is normalized to one. Further trials are then expressed as a percentage of the starting climbing index. This scoring method is useful for experiments in which similar genetic backgrounds are being subjected to varying treatments.

### Cardiac Pacing

Flies were connected to a square-wave stimulator and subjected to external electrical pacing [42]. Flies' hearts are accelerated to 6 Hz (about twice the normal speed) for 30 seconds and then scored as either a fibrillation, an arrest or a non-event. Percent fibrillation, percent arrest, and percent of arrested hearts that later recover within two minutes after the test were charted.

### Activity Monitoring

The *Drosophila* Activity Monitoring System (DAMSystem 3.0, TriKinetics Inc.) was used to track the flies' activity rate over time. The DAM2 monitor system measures individual 32 flies simultaneously, which are each placed in separate tubes that have a cotton stopper on one and food and a plastic cap on the other end. As a fly walks back and forth from one side of the tube to the other end, its passage is detected and counted by an infra-red beam which bisects the tube, and the accumulated count totals are reported to the host computer at the conclusion of each reading period. Flies were kept in the tubes for 2 hour intervals at each time point. Experiments were performed at the same time of day in each case to control for the influence of circadian rhythms.

### Aconitase Assay

Aconitase activity was assayed by using Bioxytech Aconitase-340™ Spectrophotometric Assay kit from OxisResearch™. Ten 3 week old *y^1^w^1^* Baylor male flies were homogenized in 50 ul of trisodium citrate in Tris HCl, pH 7.4 for 20 seconds and centrifuged at 800*×*g for 10 minutes. Samples were sonicated for 20 seconds and further diluted with Tris HCl, pH 7.4 to reach a concentration of 250 ug/ml. 200 ul of Tris HCl, Trisodium citrate, Isocitrate dehydrogenase, and NADP+ were added to 200 ul of the sample and Aconitase activity was measured by monitoring the production of NADPH from NADP+ at 340 nm for 5 minutes at 37°C.

### Statistical Methodology

*See* *Methods S1*

## Results

### Power Tower

*Drosophila* exhibit stereotyped, instinctive negative geotaxis behavior when dislodged to the bottom of a vial [43]. The speed at which they climb the wall of the vial after being knocked to the bottom has been commonly used as an assay for locomotor ability, and has been shown to decline with age [44], [45].

**Figure 1:**
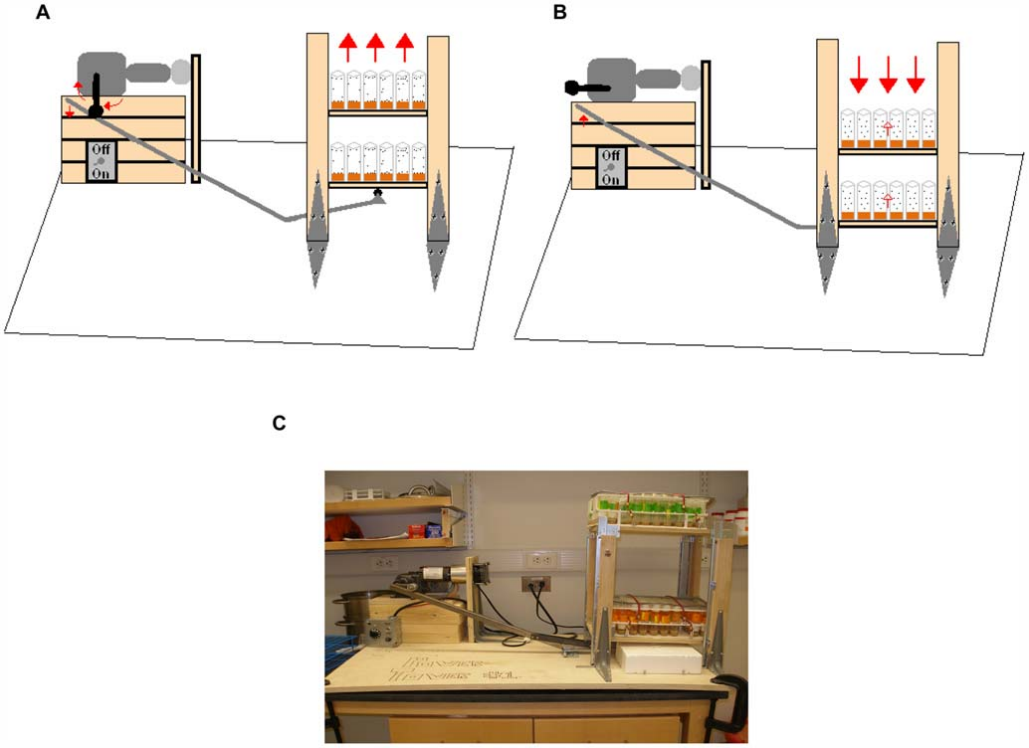
Schematic representation of the Power Tower, a motor-operated enforced climbing apparatus that exercise-trains *Drosophila*. (A): As the rotating arm circles around in a clockwise direction, the lever pushes down and the two platforms of vial holders rise up. (B): As the rotating arm rolls off, the lever lifts and the platform of vial holders drops down. The flies drop to the bottom of the vial, inducing their negative geotaxis instinct. The flies continue to repeat this process until the motor is shut off. Exercise-training thus occurs through continuous climbing. (C): An image of the Power Tower.

In order to provoke physiological responses in flies analogous to those produced in vertebrates during endurance exercise-training, we employed a device known as the Power Tower (Figure 1). The Power Tower consists of a platform that raises, then drops, several racks of fly vials simultaneously (Movie S1, Movie S2). This induces the flies' instinct for negative geotaxis and causes them to run up the side of the vial. A rotating wheel and a roller-arm allow the machine to repeat this process indefinitely until the machine is shut off. This facilitates user control of the duration and frequency of exercise.

In order to determine the exercise program with the maximal benefit, we subjected *y^1^w^67c23^* flies to 9 different exercise-training regimens with varying frequency, varying duration and different schedules of “ramping”, in which the duration of each bout of exercise is gradually increased during the experimental timecourse (Table 1). In all cases, flies were placed on the training regimen at three-four days of age, and trained for three weeks, then taken off the training for two more weeks, in order to ascertain if changes to climbing ability would persist after cessation of training. The effectiveness of various regimens was assessed by charting a “climbing index” for each vial of 20 flies on a longitudinal basis throughout the experiment (Methods S1). For each regimen, exercise-trained flies were compared to unexercised flies. Unexercised flies were placed on the Power Tower, but with a sponge stopper lowered into the vial to prevent the flies from climbing.

**Table 1:** Exercise-training regimens varying duration and bout number.

Regimens	Week	Monday	Tuesday	Wednesday	Thursday	Friday	Results
**Regimen 1**	**Week 1**	0.25	0.5	0.75	1	1.25	p-value = 0.0515
	**Week 2**	1.5	1.75	2	2.25	2.5	
	**Week 3**	2.75	3	3.25	3.5	3.75	
**Regimen 2**	**Week 1**	0.25/0.25	0.5/0.5	0.75/0.75	1/1	1.25/1.25	p-value = 0.1312
	**Week 2**	1.5/1.5	1.75/.75	2/2	2.25/2.25	2.5/2.5	
	**Week 3**	2.75/2.75	3/3	3.25/3.25	3.5/3.5	3.75/3.75	
**Regimen 3**	**Week 1**	0.5	0.5/0.5	0.5	0.5/0.5	1	p-value = 0.8572
	**Week 2**	1.5	1.5/1.5	1.5	1.5/1.5	2	
	**Week 3**	2.5	2.5/2.5	2.5	2.5/2.5	3	
**Regimen 4**	**Week 1**	0.5	0.5	0.75	0.75	1	p-value = 0.0776
	**Week 2**	1.5	1.5	1.75	1.75	2	
	**Week 3**	2.5	2.5	2.75	2.75	3.0	
**Regimen 5**	**Week 1**	0.25	0.5	0.75	1	1.25	p-value = 0.9563
	**Week 2**	1.5	1.75	2	2.25	2.5	
	**Week 3**	2.75	3	3.25	3.5	3.75	
**Regimen 6**	**Week 1**	0.5	0.5	0.5	0.5	1	p-value = 0.6470
	**Week 2**	1.5	1.5	1.5	1.5	2	
	**Week 3**	2.5	2.5	2.5	2.5	3	
**Regimen 7**	**Week 1**	-	-	-	-	2.0	p-value = 0.6384
	**Week 2**	-	-	-	-	2.5	
	**Week 3**	-	-	-	-	3.0	
**Regimen 8**	**Week 1**	2.0	-	2.0	-	2.0	p-value = 0.0036
	**Week 2**	2.5	-	2.5	-	2.5	
	**Week 3**	3.0	-	3.0	-	3.0	
**Regimen 9**	**Week 1**	**2.0**	**2.0**	**2.0**	**2.0**	**2.0**	**p-value = 0.0359**
	**Week 2**	**2.5**	**2.5**	**2.5**	**2.5**	**2.5**	
	**Week 3**	**3.0**	**3.0**	**3.0**	**3.0**	**3.0**	

Columns 3–7 represent the number of hours the flies were exercised on each day. Multiple numbers indicate that flies were subjected to more than one training bout on such days. Column 8 represents p-value for multivariate regression (treatment-by-age) of the negative geotaxis scores for exercised flies compared internally to unexercised controls. Altering the ramping schedule by changing the length of exercise bouts had a highly significant effect on the age-dependent slope of negative geotaxis (hours of exercise-by-age, p<.0001). Altering the number of bouts of exercise did not produce a significant effect (bouts-by-age, p = .7694).

As has been found in exercise experiments using aging humans [7], [8], [46], none of the exercise regimens was able to completely halt age-related decline in mobility. Several regimens, however, were able to significantly reduce the slope of age-related decline in climbing ability (Table 1). Altering the duration of exercise was critically important to maximization of negative geotaxis ability across ages, whereas altering the number of exercise repetitions did not change the outcome significantly (Table 1, hours of exercise-by-age, p<.0001). One successful training regimen (bolded in Table 1) was selected for further testing.

**Figure 2:**
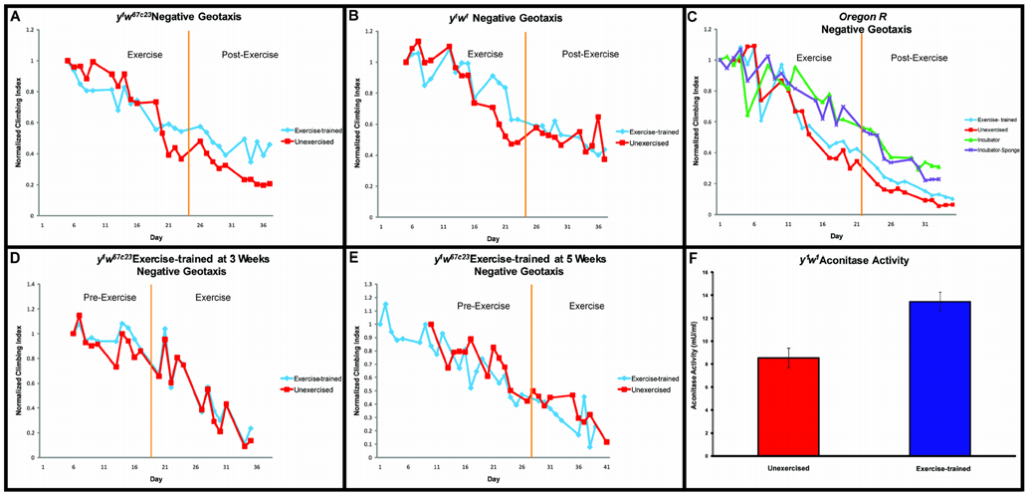
Negative geotaxis in aging, exercise-trained flies. (A): *y^1^w^67c23^* exercise-trained flies (blue diamonds) improve their negative geotaxis ability over their unexercised controls (red squares) by day 21 of the exercise-training regimen. This difference persists two weeks after cessation of exercise (multivariate regression, treatment-by-age (days 21–37): p = 0.0747; treatment effect (days 21–37) p<0.001). (B): *y^1^w^1^* exercise-trained flies (blue diamonds) demonstrate improvement in negative geotaxis from day 19 to 37 of the training regimen (multivariate regression, treatment effect: p<0.0001). (C): *Oregon R* exercised-trained flies (blue diamonds) improve their negative geotaxis ability over unexercised controls (red squares). A difference in climbing ability becomes apparent at day 17 of the time-course and this improvement over controls persists for at least two weeks after cessation of exercise (multivariate regression, treatment effect (days 17–33): p<0.001). Untreated flies (green triangles) and untreated flies with restricted movement (purple X) show no significant difference in negative geotaxis ability (multivariate regression, treatment effect: p = 0.3018). (D): *y^1^w^67c23^* flies that began exercise-training at 3 weeks of age (blue diamonds) show no significant difference in climbing ability over unexercised flies (red squares) (multivariate regression, treatment-by-age: p = 0.9893). (E): *y^1^w^67c23^* flies that began exercise-training at 5 weeks of age (blue diamonds) show no significant differences in climbing ability over control flies (red squares) (multivariate regression, treatment-by-age: p = 0.9545). (F): Aconitase activity in total protein extract from whole flies is increased following three weeks of exercise (red) as compared to isogenic, age-matched controls (blue) (t-test, p = .037).

A training paradigm of five bouts of exercise per week, with a ramped schedule of increasing duration, was retested on *y^1^w^67c23^* flies, and additionally tested on two other genotypes, *y^1^w^1^* and *Oregon R* (Fig. 2A,B,C). Although these divergent genetic backgrounds showed a varying degree of response to the training program, all three showed a statistically significant improvement in mobility (multivariate regression - *y^1^w^67c23^*: Treatment [days 21–37] p<0.001, *y^1^w^1^*: Treatment [days 19–37]: p<0.0001), *Oregon R*: Treatment [days 17–33]: p<0.001]), indicating that the training method is generally applicable and not specific to one genotype. On the other hand, the difference in the magnitude of response in different genetic backgrounds indicates that genetic variation plays a major role in modulating exercise response in flies, as it does in vertebrates. Which loci are of importance to regulate this phenomenon will be an exciting source of inquiry in upcoming years.

In order to assess potential unintended effects of treatment on the Power Tower machine, an additional control was carried out on *Oregon R* flies. In addition to the standard unexercised flies, placed on the machine with a stopper to prevent running, another cohort was kept in the incubator and scored for climbing at identical times, but never placed on the machine at all. A fourth cohort was kept in the incubator throughout the experiment, but had a sponge stopper placed in their vials during the same time the others were on the Power Tower. Flies that were never placed on the training machine had a higher climbing index across ages than did those on the Power Tower (multivariate regression, machine effect, p<0.0001 Fig. 2C), suggesting that the force of the repeated drops by the machine may be exerting damaging effects on the flies. However, this negative trend may be specific to mobility since there is no difference in percent arrest rate in the unexercised flies compared to flies that are never placed on the machine (data not shown). In summary, exercise-training improves the climbing ability of multiple genotypes in comparison to unexercised flies on the machine at the same time. The sponge stopper treatment had no measurable effect on flies' climbing response, as flies kept off the machine with a stopper had climbing indexes that were not significantly different from flies kept off the machine without a stopper (multivariate regression, stopper effect, p = 0.3018, Fig. 2C).

### Exercise during aging

Aging vertebrates have been shown to retain the ability to respond to exercise with changes in muscle size and fiber composition [47], [48] but it is unclear whether *Drosophila* can respond to exercise at advanced ages, or whether age-related declines in this model are irreversible. In order to test this directly, we raised genetically identical, age-matched cohorts of *y^1^w^67c23^* flies and subjected them to exercise-training at various ages. Despite the fact that this same genotype repeatedly demonstrates a dramatic increase in climbing ability when exercised early in life (Fig. 2A), exercise-training later in life showed no discernible effect. Cohorts that began exercising at three weeks or five weeks of age were not significantly different in their rate of age-related mobility decline compared to unexercised cohorts on the machine at the same time (multivariate regression; three-week, treatment-by-age: p = 0.9893; five week, treatment-by-age: p = 0.9545, Fig. 2D,E).

### Mitochondrial activity

In order to examine whether flies experience an increase in mitochondrial activity in response to exercise-training, we measured aconitase activity levels on total protein extracts from exercise-trained and unexercised flies of the same age and genotype. Aconitase activity was 30% higher in flies following a three-week course of exercise-training (t-test, p = .037, Fig. 2F).

### Exercise and cardiac senescence

Endurance exercise has long been known to benefit cardiac performance in patients with a variety of pathologies, including those experiencing heart failure [49]. Regular exercise also improves cardiac resistance to damage from ischemia and reperfusion [50]–[52]. Less well understood is the role of exercise in slowing non-pathological declines in cardiac performance as a result of normal aging. Many studies have examined exercise-induced cardioprotection in young animals [53] and some have evaluated exercise-induced cardioprotection in senescent animals [54]. Here, we examine cardiac stress response in flies across ages both during and following a course of exercise-training.

*y^1^w^67c23^* flies were exercised from three days of age to three weeks of age. Before, during, and after exercise-training, they were subjected to a non-invasive cardiac pacing assay [55], and charted for the percentage of flies that responded to pacing by entering a fibrillation or arrest event. Those flies that experienced cardiac arrest were observed for two minutes after stimulus to assess whether they recovered functional heartbeat spontaneously.

**Figure 3:**
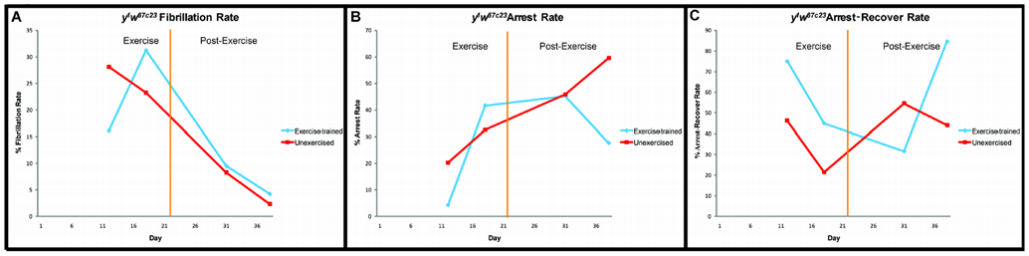
Cardiac stress resistance tests for exercise-trained and unexercised flies. (A): There is no significant difference in fibrillation rate of *y^1^w^67c23^* flies after cardiac electrical pacing between exercised (blue diamonds) and unexercised flies (red squares) (multivariate regression, treatment-by-age: p = 0.1947, chi-square = 1.682). (B): *y^1^w^67c23^* cardiac arrest rate in response to electrical pacing is shown for exercise and unexercised flies across five weeks. Exercise-trained flies (blue diamonds) display a lower arrest rate than unexercised flies (red squares) by five weeks (t-test, p = 0.001). (C): *y^1^w^67c23^ exercise*-trained flies (blue diamonds) have a higher arrest-recovery rate at five weeks than unexercised flies (red squares) (t-test, p = 0.008). All lines represent straight connective lines between data points, and are not fitted to a best-fit model.

*y^1^w^67c23^* flies showed a significant difference in fibrillation rate in response to pacing (Fig. 3A), but showed a statistically significant improvement in rate of pacing-induced arrest at advanced ages (t-test at five weeks, *y^1^w^67c23^*, p = .001, Fig. 3B). Furthermore, among those that experienced arrest, a significantly higher percentage was able to recover a normal heartbeat within two minutes (t-test at five weeks, *y^1^w^67c23^*, p = .008, Fig. 3C).

In contrast to genetic interventions that impede cardiac senescence [55], [56], [57] exercise-training does not show an immediate effect when implemented. Rather, age-related increase in arrest rate proceeds in the same way as in controls initially, then reverses course. Experiments using fewer time points performed on additional genotypes have produced similar results (data not shown).

### Diet/exercise interaction

In order to test the relationship between dietary restriction and exercise-training, we utilized a strain of *y^1^w^1^* flies that we confirmed to respond to dietary restriction by extending its lifespan (data not shown). The same strain has also been shown to improve its mobility following exercise-training (Fig. 2B). Separate cohorts of flies were subjected to exercise-training by our standard protocol, while being fed on various diets. All flies were grown to adulthood on standard 10% yeast/sucrose diet to avoid potential developmental effects, then placed on experimental diets as adults. Each diet contained 10% sucrose, while the amount of yeast was varied in each.

**Figure 4:**
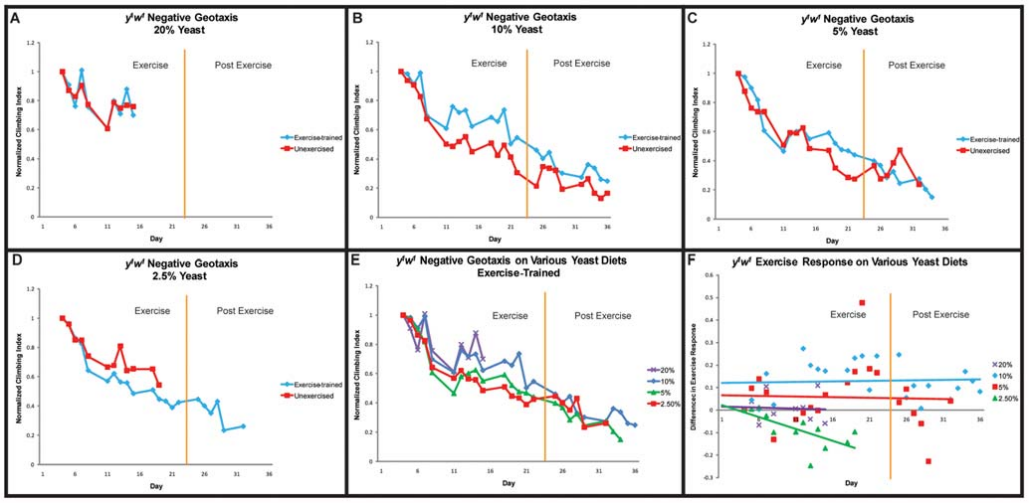
Influence of dietary yeast on negative geotaxis of aging, exercise-trained flies. (A): On a 20% yeast, 10% sucrose diet, both exercise-trained flies (blue diamonds) and unexercised *y^1^w^1^* flies (red squares) exhibit high climbing ability. (B): On the standard 10% yeast, 10% sucrose diet, *y^1^w^1^* exercise-trained flies (blue diamonds) improve their negative geotaxis ability over unexercised controls (red squares) by day 11 of the regimen and this difference persists two weeks after cessation of exercise-training (multivariate regression, treatment effect (days 11–36): p<0.0001). (C): On a 5% yeast, 10% sucrose diet, exercise-trained flies (blue diamonds) show a smaller, yet still significant improvement in negative geotaxis ability over unexercised controls (red squares) (multivariate regression, treatment effect (days 15–29): p = 0.0575). (D): On a 2.5% yeast, 10% sucrose diet, exercise-trained flies (blue diamonds) have a reduction in their negative geotaxis ability compared to unexercised flies (red squares) (multivariate regression, treatment effect: p<0.0001). (E): Negative geotaxis ability of exercise-trained *y^1^w^1^* flies are re-graphed by diet (purple Xs: 20% yeast; blue diamonds: 10% yeast; green triangles: 5% yeast; red squares: 2.5% yeast). Slope of decline in negative geotaxis ability is dependent upon the amounts of yeast in the diet (multivariate regression, diet-by-age: p = 0.0573). (F): Difference in climbing index score between exercise-trained and unexercised flies on various yeast diets.

Flies kept on 20% yeast, twice the normal amount in our standard diet, displayed an unusually high mobility, whether exercised or unexercised (Fig. 4A), implying that nutritional components found in yeast (principally protein and lipid) may be limiting for climbing ability in adult flies. This highly enriched diet causes a reduced lifespan in unexercised flies [58, 59, data not shown], and exercise-training did not rescue this effect. Indeed, all 100 flies in the cohort had died by day 15 of the experimental timecourse. Flies kept on 10% yeast, the standard laboratory diet, showed a similar response to exercise as was seen in prior experiments (Fig. 4B,2B).

Flies on 5% yeast, in our hands (data not shown), display a significant lifespan extension as compared to the standard 10% yeast diet. Such dietary restricted flies do not, however, display any reduction in age-related mobility decline [60]. When subjected to exercise-training, flies on 5% yeast respond with a significantly improved mobility during the exercise course (multivariate regression, treatment effect (days 15–29): p = 0.0575, Fig. 4C), suggesting that, although dietary restriction alone cannot improve this aspect of functional senescence, dietary restriction in combination with a regular program of exercise can provide some of the benefits of both. However, unlike the same flies on 10% yeast, flies on 5% yeast did not persist to show improved mobility after the termination of the exercise program (compare Fig. 4B with 4C).

Flies on 2.5% yeast exhibit a reduced lifespan (data not shown), possibly due to malnutrition. Under these dietary conditions, exercise-training results in a reduction in climbing ability and a hastening of age-related mobility decline (Fig. 4D), most likely due to an insufficient energy intake.

When the mobility of exercise-trained flies across diets is compared directly, it seems clear that the degree of dietary yeast is correlated with climbing speed (multivariate regression, diet-by-age: p = 0.0573, Fig. 4E), as following two weeks of regular exercise, the mobility of flies on different diets shows the same rank order as the yeast concentration. This ceases to be the case after exercise is discontinued, however, as post-exercise mobility rapidly becomes similar again regardless of diet (Fig. 4E).

A difference plot between exercised and unexercised flies on each diet (Fig. 4F) reveals that differences are seen very early following induction of exercise and once the difference in mobility manifests itself, it remains relatively constant throughout the course of exercise, suggesting that long-term changes, rather than gradual changes, are induced on a short time-scale by exercise in flies. Also evident from this difference plot is that although the highest degree of climbing speed is achieved with 20% yeast, the biggest exercise-induced change in climbing speed comes at 10% yeast, with 5% yeast also showing a significant increase. Flies on 20% yeast exhibit a similar high climbing speed whether exercised or not, while flies on 2.5% yeast actually become slower climbers when exercised (Fig. 4F).

In order to examine the role of dietary carbohydrate levels in modulating the exercise response in flies, we exercise-trained *y^1^w^1^* flies while feeding them on 2.5%, 5% or 20% sucrose. The yeast concentration was held steady at 10% throughout.

**Figure 5:**
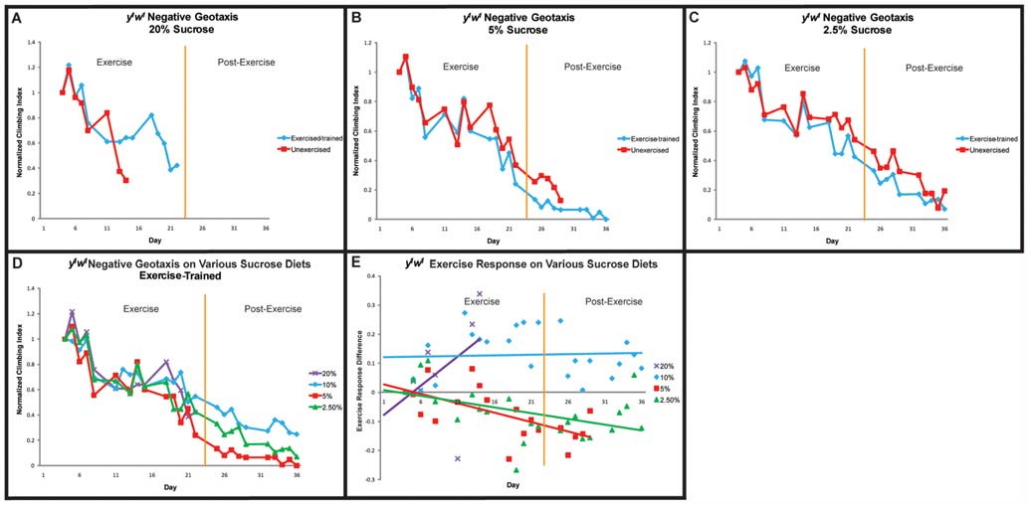
Negative geotaxis is shown for *y^1^w^1^* while changing the amount of sucrose in the diet and keeping yeast levels constant. (A): On a 20% sucrose, 10% yeast diet, no apparent difference is seen early on in the time-course of exercise-training. Lifespan was greatly reduced on this diet, therefore too small of a sample size was available for statistical analysis. (B): On a 5% sucrose, 10% yeast diet, exercise-trained flies (blue diamonds) have a reduction in negative geotaxis ability compared to unexercised controls (red squares) (Treatment (days 19–29): p<0.0001). (C) On a 2.5%, 10% yeast diet, exercise-trained flies (blue diamonds) have a decline in negative geotaxis ability compared to unexercised controls (red squares) (Treatment (days 19–36): p = 0.0088). (D): Negative geotaxis ability of exercise-trained *y^1^w^1^* flies are graphed by diet (purple Xs: 20% sucrose; blue diamonds: 10% sucrose; green triangles: 5% sucrose; red squares: 2.5% sucrose). (E): A difference plot between exercise-trained and unexercised flies on various sucrose diets. Data for 10% yeast, 10% sucrose diet is derived from the experiment shown in Figure 4B and is reused for comparative purposes.

On 20% sucrose, twice the amount of sugar as in our standard laboratory diet, little difference in mobility was seen during the first two weeks of exercise training (Fig. 5A). Lifespan is greatly reduced on this enriched diet (data not shown). After two weeks of exercise, the sample size of surviving flies became too low for reliable statistical analysis, and after three weeks, all flies in the cohort had died.

On 5% sucrose, half the amount of sugar in our standard diet, no difference in mobility was observed during the training course (Fig. 5B), although a small, but statistically significant reduction in mobility among the exercised cohort was observed in the two weeks immediately after training was halted (multivariate regression, treatment effect (days 19–29): p<0.0001, Fig. 5B).

On 2.5% sucrose, a level of carbohydrate intake that results in a reduced lifespan, the exercised cohort experienced a small, but significant increase in its age-related mobility decline, in comparison with unexercised controls (multivariate regression, treatment effect (days 19–36): p = 0.0088, Fig. 5C).

When comparing mobility of exercise-trained flies across different percentages of sucrose in the diet (Fig. 5D), there is not quite as clear of a distinction between the amount of sucrose available to flies and their climbing performance as there is when examining different amounts of yeast (Fig. 4E).

A difference plot reveals that reduced sucrose diets actually cause a mild loss of climbing speed during exercise training, whereas flies on the standard laboratory 10% diet respond early by improving climbing speed and maintain that difference throughout and following exercise (Fig. 5E). Although we cannot detect any linear relationship between sucrose levels and response to exercise, it seems that a minimum amount of dietary sugar is required for exercise to produce improvements in climbing speed.

Taken together, these results indicate that dietary yeast plays a much larger role in modifying the response to exercise-training than does dietary sugar. In the food mixture used in this study, yeast constitutes the only source of both protein and lipid. Both of these components have been proposed to play a role in the regulation of lifespan [58], [59] and exercise response [61], [62]. Future experiments will examine in more detail whether protein, lipid or both are critical to this response in flies.

### Mitochondrial Efficiency and Exercise-Training

Changes in mitochondrial activity and efficiency are known to result from endurance exercise-training in multiple species [63], [64]. Such changes are thought to contribute to beneficial alterations in the metabolic state of exercised animals.

A fly line selectively bred for longevity over many generations (designated La) exhibits an improved mitochondrial efficiency [65] in comparison to its wild-caught, isogenized progenitor line (designated Ra). La and Ra flies were exercise-trained and tested for mobility during three weeks of exercise and for two weeks post-exercise.

**Figure 6:**
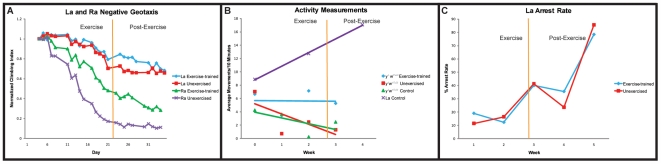
Mito-efficient flies display improved mobility. (A): Negative geotaxis of aging, exercise-trained, La flies. Ra exercise-trained flies (green triangles) display improvement in climbing index at day 7 of the exercise-training regimen and this difference persists two weeks after cessation of exercise-training compared to the unexercised controls (purple Xs) (multivariate regression, treatment, p<0.0001; treatment-by-age: p = 0.0023). In contrast, La flies show a slower age-related decline in mobility compared to Ra flies (La exercise-trained (blue diamonds) versus Ra exercise-trained (green triangles): (multivariate regression, genotype-by-age: p<0.0001). (B): Activity rate is measured as number of times crossing a beam threshold per 10 minutes during 2-hour intervals. *y^1^w^67c23^* exercise-trained flies (blue diamonds) have higher activity rates over 4 weeks compared to *y^1^w^67c23^* unexercised flies (red squares) (multivariate regression, treatment-by-age: p = 0.0365) and *y^1^w^67c23^* control flies (green triangles) not placed on the Power Tower. Unexercised flies placed on the machine and control flies not placed on the machine are not significantly different from each other (multivariate regression, treatment-by-age: p = 0.2237). La flies (purple Xs) not placed on the machine have a much higher activity rate throughout 4 weeks compared to the wildtype. (C): Arrest rate for La exercise-trained flies (blue diamonds) is not significantly different from La unexercised flies (red squares) across 5 weeks (multivariate regression, treatment: p = 0.8688, treatment-by-age: p = 0.3784).

The mitochondrial-efficient La line displayed extraordinarily high mobility both before and during exercise-training (Fig. 6A). Despite the already high mobility of these flies, a small, statistically significant, improvement occurred following the exercise course as compared to unexercised La flies (multivariate regression, treatment effect: p<0.0001). By contrast, the progenitor Ra flies displayed a low pre-exercise mobility, similar to other wild-type genetic backgrounds (compare Fig. 6A to Fig. 2C). Exercise-training, however, produced a much more dramatic reduction in age-related mobility decline in Ra flies than in La flies (multivariate regression, treatment effect, p<0.0001; treatment-by-age: p = 0.0023, Fig. 6A).

In cold-blooded invertebrate species, an increase in mitochondrial activity can be reflected by an increase in baseline activity levels. Consistent with this idea, exercised *y^1^w^67c23^* flies displayed a significant impedance of age-related decline in baseline activity, as measured by longitudinal single-fly activity monitoring (multivariate regression, treatment-by-age: p = 0.0365, Fig. 6B). In these experiments, the amount of movement exhibited by flies in a defined period of time is measured. Unlike the negative geotaxis assay, no inducement is given to flies to provoke movement. Baseline activity was measured at the same time each day to control for potential circadian effects, and was measured prior to daily exercise to eliminate potential effects of fatigue. In this context, unexercised La flies also behave as if they had been exercised, displaying a high activity rate that persists throughout the first three weeks of age, after wild-type, unexercised flies have experienced a significant reduction in activity (Figure 6B).

We also subjected mitochondrial-efficient La flies to cardiac stress tests once a week from one to five weeks of age. Unlike wild-type flies (Fig. 3), La flies show no difference in pacing-induced arrest rate or in arrest-recovery rate following exercise (multivariate regression, treatment: p = 0.8688, Treatment-by-age: p = 0.3784, Fig. 6C).

These results are consistent with a model in which changes in mitochondrial efficiency are a significant factor in converting exercise-training into a slower age-related mobility decline, but play little or no role in converting exercise-training into slower age-related decline in cardiac stress resistance. The genetic and cellular mechanisms responsible for the effect of exercise on cardiac aging remain to be elucidated.

## Discussion

### Drosophila as an exercise-training model

Controlled programs of exercise have long been known to provide a wide spectrum of physiological benefits to aging humans and other animals. However, the relationship between controlled, regular exercise and the rate of change in mobile capacity that comes about with aging is still not well understood. Furthermore, although progress has been made in identifying genetic factors involved in tissue-specific responses to exercise, especially in skeletal muscle [66]–[68] it is likely that additional potentially therapeutic genetic factors remain to be identified.

Most vertebrate studies, including those with humans as subjects, have employed cross-sectional study designs, due to the high cost and expense of doing longitudinal studies on long-lived species. Rarely have such studies employed multiple genetic backgrounds or inter-crossed animals. Invertebrate studies on exercise during aging have been even fewer, in large part due to the dearth of available physiological assays suitable for smaller model species, such as flies and worms.

Here we present a novel exercise-training device for *Drosophila*, suitable for both large isogenized populations and longitudinal experimental designs. The effects of exercise can be evaluated by a suite of non-invasive assays for mobility and cardiac performance. Since age-related declines in both mobility and cardiac function occur almost entirely within the first five weeks of life [42], [44], [45], [55], [58], [60] use of *Drosophila* allows rapid testing of genetic or environmental interventions. The public availability of mutations covering most of the *Drosophila* genome is also conducive to rapid screening for novel loci that modulate the physiological response to exercise.

Prior experiments using insect models to examine the relationship of exercise to aging have utilized continuous increase in flying activity as an exercise stimulus. Under these conditions, exercise was shown to increase oxidative stress, as well as resulting oxidative damage, and to decrease lifespan [35], [36]. We have instead employed running as an exercise stimulus. Since studies on human athletes and vertebrate models have indicated that controlled exercise programs of gradually increasing duration are more beneficial than continuous high levels of exercise [69], we have utilized a gradual, ramped program of training in the fly model system. We have identified a particular exercise program that provides several benefits that are reminiscent of the physiological changes seen in vertebrates following exercise-training, 1) increased performance in climbing/running ability, 2) increase in cardiac stress resistance, 3) increase in mitochondrial activity. These similarities strongly suggest that the changes seen in *Drosophila* during exercise-training are conserved, and justify the fly as a model system for further study of exercise-based anti-aging mechanisms.

### Exercise slows age-related functional decline

Young flies that are exposed to a regular exercise course display a significant reduction in age-related functional declines, both in mobility and in cardiac stress resistance. Interestingly, in both functions that we measured during the aging process, improvement persisted after exercise was halted, indicating that long-term changes are induced by this training program.

Some differences exist in the two responses assayed, however. The mobility of exercised flies begins to diverge from that of unexercised controls rapidly after induction of exercise. Indeed, the pattern of mobility decline seen in exercised flies is consistent with a delayed onset of age-related symptoms, as the mobility of exercised flies declines at a similar rate to that of unexercised flies, once the decline begins (Fig. 2).

By contrast, cardiac stress resistance appears to decline at the same rate as unexercised controls for the first three weeks of life, while exercise-training is in progress. It is later, at four and five weeks of age, that a difference appears, as exercised flies halt their decline in stress resistance, and show dramatic improvements in both arrest rate and in ability of arrested hearts to regain normal function (Fig. 3). These patterns are likely to be generally applicable, as they are seen in multiple genetic backgrounds consistently. The difference in the pattern of response in these physiological assays suggests that either exercise-induced improvement in different organ systems comes about through multiple mechanisms, or that the same mechanisms act on different time-scales in different tissues. We favor the former possibility, since a genetic model for increased mitochondrial efficiency, the La fly, exhibits potent effects on mobility without any demonstrable effect on cardiac senescence.

Exercise is known to produce acute benefits to the physiology of aging humans, and to improve symptoms associated with age-related pathologies [70]. In flies measured longitudinally across ages, however, we see no substantial slowing of age-related decline following late induction of exercise (Fig. 2). One possible explanation for this is that the exercise course most capable of producing long-term physiological changes is quite a strenuous one. It may be that older flies are not capable of executing such a program without sustaining damage that does as much harm as good.

An alternate explanation is that the age-related decline in flies' negative geotaxis response and resistance to cardiac stress is largely complete by three weeks of age, despite the fact that this age is less than the mean lifespan for these genetic backgrounds. This interpretation is consistent with previous experiments in which the slope of age-related decline in negative geotaxis [45] and cardiac stress resistance [55]–[57] is, in fact, highest between one and three weeks of age, after which a leveling off occurs prior to the population entering a log phase of mortality.

While changes in diet are capable of producing instant, acute, and reversible effects on mortality [71], we see no evidence that exercise affects functional aging in the same way. Rather, it seems that an early, regular program of exercise during young adulthood creates long-term physiological changes that produce substantial benefits to late-life function. Once substantial decline has occurred, exercise-training is not effective at reversing the trend.

### Exercise and cardiac senescence

During the process of normal aging, cardiac function in *Drosophila* undergoes measurable changes in several aspects of performance [72]. A particularly sensitive assay for physiological age in fly cardiac function is external electrical pacing [42]. Temporary acceleration of heart rate causes some percentage of flies to undergo either fibrillation or arrest events. The likelihood that an individual fly will experience such an event is highly age-dependent, and the incidence of both fibrillation and arrest increases dramatically with age, while the percentage of flies able to recover from such events declines with age [55].

Genetic factors that regulate lifespan in multiple species exert tissue-autonomous effects on this measure of cardiac senescence. Both insulin signaling [55] and TOR activity [57] affect both fibrillation and arrest rates. Other factors have been shown to affect one of these factors preferentially. For example, the potassium channel *KCNQ* has profound effects on the age-related decline in rhythmicity without affecting contractile strength [56], while mutations in the fly *dystrophin* gene lead to profound abnormalities in contractile force [73].

Exercise-training has a significant, but specific, effect on the rate of cardiac senescence. The rate of arrest in response to acute stress is markedly reduced in older flies that have been exercised, in comparison to unexercised controls (Fig. 3). However, fibrillation rate is relatively unaffected. Exercise-training seems, therefore, to specifically protect against age-related declines in contractile ability and the capacity to increase contractile rate without failure, but we can detect no evidence of an effect on electrical conductivity or rhythmic regulation.

### Dietary restriction and exercise-training

The relationship between dietary restriction, exercise-training and functional aging is a complex one. Dietary restriction is known to extend maximal lifespan in multiple species, but has not been demonstrated to improve mobile capacity in aged flies. Indeed, diets that produce significant lifespan extension are unable to slow age-related decline in negative geotaxis ability [58] or in cardiac stress resistance (Morley and Wessells, unpublished). By contrast, exercise-training significantly preserves motility during aging in flies, but is not capable of extending maximal lifespan. Dietary restriction causes an immediate, acute change in mortality rate that can be reversed equally rapidly by restoring flies to their standard diet [58]. By contrast, exercise-training produces a rapid response that persists even after exercise has been stopped. These differences argue in favor of a difference in mechanism between dietary restriction and exercise-training. If these interventions act through separate mechanisms, the encouraging possibility exists that both dietary restriction and exercise-training could act in parallel to simultaneously provide the benefits of both. These results support such a possibility, as flies on a 5% yeast diet, a diet that produces significant lifespan extension, also respond robustly to exercise-training simultaneously (Fig. 4), albeit not as strongly as flies on a standard diet.

The maximal baseline mobility in flies correlates with the maximum yeast contained in the diet, consistent with a model in which acute yeast levels are a limiting factor in immediate mobile capacity. Interestingly, changes in dietary yeast have much more profound effects on the response to exercise than do changes in sugar (Fig. 4, 5). Similar results have been seen in the relationship of diet to lifespan extension, where yeast restriction alone can generate significant lifespan extension in some cases, whereas sugar/carbohydrate restriction cannot. Recent studies have also indicated that the ratio of protein to sugar in the diet may be more important to lifespan regulation than the raw amounts of either component [59]. It remains to be seen whether protein/carbohydrate ratio is also important for regulating the exercise response in flies.

### Mitochondrial activity during exercise

Changes in mitochondrial biogenesis and mitochondrial efficiency are thought to be an integral part of the conserved response to exercise in many species [75], [76], [77]. Likewise, changes in mitochondrial efficiency and levels of ROS production are thought to underlie lifespan extension via dietary restriction.

Exercise upregulates global levels of mitochondrial activity in flies, as judged by aconitase activity levels (Fig. 2F). Long-lived flies with improved mitochondrial efficiency (La) display high baseline mobility, then undergo a small further extension in mobility following exercise-training (Fig. 6). The wild-type background control flies (Ra), by contrast, begin with a much lower mobility level, then increase dramatically following exercise (Fig. 6).

These results support a model in which mitochondrial efficiency is a significant determining factor in both baseline mobility and the capacity for animals to respond to exercise-training by improving mobility. The fact that La flies still demonstrate some degree of improvement following exercise argues that, although mitochondrial changes are a critical part of the exercise response, other factors are also involved. At this time, we cannot rule out the possibility that other undiscovered differences between La and Ra flies also contribute to the phenotypes observed here.

The ability to model exercise-training in *Drosophila* opens the door to many future avenues for dissecting the response of various organ systems to exercise during the aging process. The relationship of exercise and aging to other environmental factors, such as global metabolism, diet, and genetics, can be plausibly explored using this model using both longitudinal and cross-sectional study designs.

The identification of conserved genetic factors necessary for exercise to produce physiological changes in the fly system will have implications, not only for the basic biology of aging and exercise physiology, but will coincidentally identify potential therapeutic targets that may allow the benefits of exercise to be provided to patients unable to exercise, due to injury or pathology.

## Supporting Information

Methods S1 Detailed description of construction, use, and suggested statistical methodology for the “Power Tower” Drosophila exercise-trainer
(0.03 MB DOC)

Movie S1 The rotating arm circles through clockwise pushing down the lever. As this occurs, the double platform of vial holders rises. As the arm circles through, the platforms drop.
(8.19 MB AVI)

Movie S2 As the Power Tower raises and then drops, the flies will drop to the bottom of the vial and then ascend the side of the vial due to their instinct for negative geotaxis.
(8.23 MB AVI)

## References

[1] Cohen-Mansfield J, Frank J (2008). Relationship between perceived needs and assessed needs for services in community-dwelling older persons.. Gerontologist.

[2] Espeland MA, Gill TM, Guralnik J, Miller ME, Fielding R (2007). Designing clinical trials of interventions for mobility disability: results from the lifestyle interventions and independence for elders pilot (LIFE-P) trial.. J Gerontol A Biol Sci Med Sci.

[3] Englander F, Hodson TJ, Terregrossa RA (1996). Economic dimensions of slip and fall injuries.. J Forensic Sci.

[4] Gaebler S (1993). Predicting which patient will fall again … and again.. J Adv Nurs.

[5] Sattin RW (1992). Falls among older persons: a public health perspective.. Annu Rev Public Health.

[6] Tideiksaar R (2002). Falls in Older People: Prevention and Management (3rd ed.).

[7] Buchman AS, Boyle PA, Wilson RS, Bienias JL, Bennett DA (2007). Physical activity and motor decline in older persons.. Muscle Nerve.

[8] Paterson DH, Jones GR, Rice CL (2007). Ageing and physical activity: evidence to develop exercise recommendations for older adults.. Can J Public Health.

[9] Adams V, Doring C, Schuler G (2008). Impact of physical exercise on alterations in the skeletal muscle in patients with chronic heart failure.. Front Biosci.

[10] Kemi OJ, Ellingsen O, Smith GL, Wisloff U (2008). Exercise-induced changes in calcium handling in left ventricular cardiomyocytes.. Front Biosci.

[11] Ascensão A, Ferreira R, Magalhães J (2007). Exercise-induced cardioprotection–biochemical, morphological and functional evidence in whole tissue and isolated mitochondria.. Int J Cardiol.

[12] Bauman AE (2004). Updating the evidence that physical activity is good for health: an epidemiological review.. J Sci Med Sport.

[13] Saraceni C, Broderick TL (2007). Cardiac and metabolic consequences of aerobic exercise training in experimental diabetes.. Curr Diabetes Rev.

[14] Musi N, Hayashi T, Fujii N, Hirshman MF, Witters LA (2001). AMP-activated protein kinase activity and glucose uptake in rat skeletal muscle.. Am J Physiol Endocrinol Metab.

[15] Tunstall RJ, Mehan KA, Wadley GD, Collier GR, Bonen A (2002). Exercise training increases lipid metabolism gene expression in human skeletal muscle.. Am J Physiol Endocrinol Metab.

[16] To K, Yamaza H, Komatsu T, Hayashida T, Hayashi H (2007). Down-regulation of AMP-activated protein kinase by calorie restriction in rat liver.. Exp Gerontol.

[17] Anderson RM, Barger JL, Edwards MG, Braun KH, O'Connor CE (2008). Dynamic regulation of PGC-1alpha localization and turnover implicates mitochondrial adaptation in calorie restriction and the stress response.. Aging Cell.

[18] Shi T, Wang F, Stieren E, Tong Q (2005). SIRT3, a mitochondrial sirtuin deacetylase, regulates mitochondrial function and thermogenesis in brown adipocytes.. J Biol Chem.

[19] Oberdoerffer P, Michan S, McVay M, Mostoslavsky R, Vann J (2008). SIRT1 redistribution on chromatin promotes genomic stability but alters gene expression during aging.. Cell.

[20] Sinclair DA (2005). Toward a unified theory of caloric restriction and longevity regulation.. Mech Ageing Dev.

[21] Haigis MC, Guarente LP (2006). Mammalian sirtuins–emerging roles in physiology, aging, and calorie restriction.. Genes Dev.

[22] Holloszy JO, Kohrt W (1995). Handbook of physiology.

[23] Lin SJ, Kaeberlein M, Andalis AA, Sturtz LA, Defossez PA (2002). Calorie restriction extends Saccharomyces cerevisiae lifespan by increasing respiration.. Nature.

[24] Merry BJ (2004). Oxidative stress and mitochondrial function with aging–the effects of calorie restriction.. Aging Cell.

[25] Short KR, Vittone JL, Bigelow ML, Proctor DN, Nair KS (2004). Age and aerobic exercise training effects on whole body and muscle protein metabolism.. Am J Physiol Endocrinol Metab.

[26] Soh JW, Hotic S, Arking R (2007). Dietary restriction in Drosophila is dependent on mitochondrial efficiency and constrained by pre-existing extended longevity.. Mech Ageing Dev.

[27] Guarente L (2008). Mitochondria–a nexus for aging, calorie restriction, and sirtuins?. Cell.

[28] Hollander J, Fiebig R, Gore M, Bejma J, Ookawara T (1999). Superoxide dismutase gene expression in skeletal muscle: fiber-specific adaptation to endurance training.. Am J Physiol.

[29] Bejma J, Ramires P, Ji LL (2000). Free radical generation and oxidative stress with ageing and exercise: differential effects in the myocardium and liver.. Acta Physiol Scand.

[30] Sreekumar R, Unnikrishnan J, Fu A, Nygren J, Short KR (2002). Effects of caloric restriction on mitochondrial function and gene transcripts in rat muscle.. Am J Physiol Endocrinol Metab.

[31] Dhahbi JM, Tsuchiya T, Kim HJ, Mote PL, Spindler SR (2006). Gene expression and physiologic responses of the heart to the initiation and withdrawal of caloric restriction.. J Gerontol A Biol Sci Med Sci.

[32] Barros MH, Bandy B, Tahara EB, Kowaltowski AJ (2004). Higher respiratory activity decreases mitochondrial reactive oxygen release and increases life span in Saccharomyces cerevisiae.. J Biol Chem.

[33] Tahara EB, Barros MH, Oliveira GA, Netto LE, Kowaltowski AJ (2007). Dihydrolipoyl dehydrogenase as a source of reactive oxygen species inhibited by caloric restriction and involved in Saccharomyces cerevisiae aging.. FASEB J.

[34] Wang F, Nguyen M, Qin FX, Tong Q (2007). SIRT2 deacetylates FOXO3a in response to oxidative stress and caloric restriction.. Aging Cell.

[35] Yan LJ, Sohal RS (2000). Prevention of flight activity prolongs the life span of the housefly, Musca domestica, and attenuates the age-associated oxidative damamge to specific mitochondrial proteins.. Free Radic Biol Med.

[36] Magwere T, Pamplona R, Miwa S, Martinez-Diaz P, Portero-Otin M (2006). Flight activity, mortality rates, and lipoxidative damage in Drosophila.. J Gerontol A Biol Sci Med Sci.

[37] Grotewiel MS, Martin I, Bhandari P, Cook-Wiens E (2005). Functional senescence in Drosophila melanogaster.. Ageing Res Rev.

[38] Holloszy JO, Smith EK, Vining M, Adams S (1985). Effect of voluntary exercise on longevity of rats.. J Appl Physiol.

[39] Holloszy JO (1997). Mortality rate and longevity of food-restricted exercising male rats: a reevaluation.. J Appl Physiol.

[40] Holloszy JO (1998). Longevity of exercising male rats: effect of an antioxidant supplemented diet.. Mech Ageing Dev.

[41] Masoro EJ (2005). Overview of caloric restriction and ageing.. Mech Ageing Dev.

[42] Wessells RJ, Bodmer R (2004). Screening assays for heart function mutants in Drosophila.. Biotechniques.

[43] Miquel J, Lundgren PR, Bensch KG, Atlan H (1976). Effects of temperature on the life span, vitality and fine structure of Drosophila melanogaster.. Mech Ageing Dev.

[44] Gargano JW, Martin I, Bhandari P, Grotewiel MS (2005). Rapid iterative negative geotaxis (RING): a new method for assessing age-related locomotor decline in Drosophila.. Exp Gerontol.

[45] Rhodenizer D, Martin I, Bhandari P, Pletcher SD, Grotewiel M (2008). Genetic and environmental factors impact age-related impairment of negative geotaxis in Drosophila by altering age-dependent climbing speed.. Exp Gerontol.

[46] Faulkner JA, Larkin LM, Claflin DR, Brooks SV (2007). Age-related changes in the structure and function of skeletal muscles.. Clin Exp Pharmacol Physiol.

[47] Baar K (2006). Training for endurance and strength: lessons from cell signaling.. Med Sci Sports Exerc.

[48] Favier FB, Benoit H, Freyssenet D (2008). Cellular and molecular events controlling skeletal muscle mass in response to altered use.. Pflugers Arch.

[49] Owen KL, Pretorius L, McMullen JR (2009). The protective effects of exercise and phosphoinositide 3-kinase (p110alpha) in the failing heart.. Clin Sci (Lond).

[50] Ferrara N, Pisanelli P, Voza M, Abete P, Leosco D (2002). The aging heart and exercise training.. Arch Gerontol Geriatr Suppl.

[51] Roberts CK, Barnard RJ (2005). Effects of exercise and diet on chronic disease.. J Appl Physiol.

[52] Kruk J (2007). Physical activity in the prevention of the most frequent chronic diseases: an analysis of the recent evidence.. Asian Pac J Cancer Prev.

[53] Starnes JW, Taylor RP (2007). Exercise-induced cardioprotection: endogenous mechanisms.. Med Sci Sports Exerc.

[54] LePage ML, Crowther JH, Harrington EF, Engler P (2008). Psychological correlates of fasting and vigorous exercise as compensatory strategies in undergraduate women.. Eat Behav.

[55] Wessells RJ, Fitzgerald E, Cypser JR, Tatar M, Bodmer R (2004). Insulin regulation of heart function in aging fruit flies.. Nat Genet.

[56] Ocorr K, Reeves NL, Wessells RJ, Fink M, Chen HS (2007). KCNQ potassium channel mutations cause cardiac arrhythmias in Drosophila that mimic the effects of aging.. Proc Natl Acad Sci U S A.

[57] Luong N, Davies CR, Wessells RJ, Graham SM, King MT (2006). Activated FOXO-mediated insulin resistance is blocked by reduction of TOR activity.. Cell Metab.

[58] Mair W, Piper MD, Partridge L (2005). Calories do not explain extension of life span by dietary restriction in Drosophila.. PLoS Biol.

[59] Skorupa DA, Dervisefendic A, Zwiener J, Pletcher SD (2008). Dietary composition specifies consumption, obesity, and lifespan in Drosophila melanogaster.. Aging Cell.

[60] Bhandari P, Jones MA, Martin I, Grotewiel MS (2007). Dietary restriction alters demographic but not behavioral aging in Drosophila.. Aging Cell.

[61] Rennie MJ (2007). Exercise- and nutrient-controlled mechanisms involved in maintenance of the musculoskeletal mass.. Biochem Soc Trans.

[62] Drummond MJ, Dreyer HC, Pennings B, Fry CS, Dhanani S, Dillon EL (2008). Skeletal muscle protein anabolic response to resistance exercise and essential amino acids is delayed with aging.. J Appl Physiol.

[63] Gollnick PD, Saltin B (1982). Significance of skeletal muscle oxidative enzyme enhancement with endurance training.. Clin Physiol.

[64] Davidson SR, Burnett M, Hoffman-Goetz L (2006). Training effects in mice after long-term voluntary exercise.. Med Sci Sports Exerc.

[65] Arking R, Buck S, Novoseltev VN, Hwangbo DS, Lane M (2002). Genomic plasticity, energy allocations, and the extended longevity phenotypes of Drosophila.. Ageing Res Rev.

[66] Mahoney DJ, Parise G, Melov S, Safdar A, Tarnopolsky MA (2005). Analysis of global mRNA expression in human skeletal muscle during recovery from endurance exercise.. FASEB J.

[67] Benton CR, Yoshida Y, Lally J, Han XX, Hatta H (2008). PGC-1alpha increases skeletal muscle lactate uptake by increasing the expression of MCT1 but not MCT2 or MCT4.. Physiol Genomics.

[68] Drummond MJ, Rasmussen BB (2008). Leucine-enriched nutrients and the regulation of mammalian target of rapamycin signalling and human skeletal muscle protein synthesis.. Curr Opin Clin Nutr Metab Care.

[69] Food and Nutrition Board and Institute of Medicine (2005 (released 2002)). Dietary Reference Intakes for Energy, Carbohydrate, Fiber, Fat, Fatty Acids, Cholesterol, Protein, and Amino Acids (Macronutrients).

[70] Carter CS, Hofer T, Seo AY, Leeuwenburgh C (2007). Molecular mechanisms of life- and health-span extension: role of calorie restriction and exercise intervention.. Appl Physiol Nutr Metab.

[71] Mair W, Goymer P, Pletcher SD, Partridge L (2003). Demography of dietary restriction and death in Drosophila.. Science.

[72] Wessells RJ, Bodmer R (2007). Cardiac Aging.. Semin Cell Dev Biol.

[73] Taghli-Lamallem O, Akasaka T, Hogg G, Nudel U, Yaffe D (2008). Dystrophin deficiency in Drosophila reduces lifespan and causes a dilated cardiomyopathy phenotype.. Aging Cell.

[74] Lee KP, Simpson SJ, Clissold FJ, Brooks R, Ballard JWO (2008). Lifespan and reproduction in Drosophila: New insights from nutritional geometry.. PNAS.

[75] López-Lluch G, Irusta PM, Navas P, de Cabo R (2008). Mitochondrial biogenesis and healthy aging.. Exp Gerontol.

[76] Handschin C, Choi CS, Chin S, Kim S, Kawamori D (2007). Abnormal glucose homeostasis in skeletal muscle-specific PGC-1alpha knockout mice reveals skeletal muscle-pancreatic beta cell crosstalk.. J Clin Invest.

[77] Menshikova EV, Ritov VB, Ferrell RE, Azuma K, Goodpaster BH (2007). Characteristics of skeletal muscle mitochondrial biogenesis induced by moderate-intensity exercise and weight loss in obesity.. J Appl Physiol.

