# Supplementary material for: Exercise-Training in Young Drosophila melanogaster Reduces Age-Related Decline in Mobility and Cardiac Performance
Source: PLoS One. 2009 Jun 11;4(6):e5886. doi: 10.1371/journal.pone.0005886 (PMC2691613; doi:10.1371/journal.pone.0005886)
Supplement: Methods S1 — Detailed description of construction, use, and suggested statistical methodology for the “Power Tower” Drosophila exercise-trainer (0.03 MB DOC) [file pone.0005886.s001.doc]

Supplemental Materials and Methods

Exercise-Training Machine Construction

The Power Tower sits on a rectangular piece of plywood that is clamped to a table. A 4 or 4.5 rpm gear motor from Grainger rests on top of 4 1-inch slabs of plywood. The motor, an AC/DC speed control, a fuse, and an on/off switch are wired together into a juncture box. A 3-inch fan from Radio Shack is drilled into a ½ inch piece of plywood standing vertically and connected to a 3-inch diameter metal cylinder to cool the motor. A custom-made rotating arm is connected to the motor, which spins in a clockwise direction, pushing down one side of a ¾ inch square tube stock. This is attached to the plywood using a door hinge. As this pushes down, a two-level platform of racks filled with fly vials pushes up. Rollers are mounted both on the rotating arm and on the end of the tube stock below the platform. As the rollers on the arm clear the tube stock, the arm snaps back up, dropping the platform back down. Both platforms of racks sit on top of a piece of plywood attached by double sided sticky tape and bungee cords that connect to screw eyes. The platforms slide up and down using drawer sliders that are attached to the plywood with brackets. The racks are lined with double-sided sticky tape and a square grid screen attached by a bungee cord that holds the vials in place. A cushion, formed from a Styrofoam base covered with layers of corkboard and rubber weatherseal, is placed below the platform to dampen the shock of the drop.

Exercise Protocol

Flies are housed in a 25^0^ C incubator with 50% humidity and a 12-hour light/dark cycle. They are fed a 10% yeast, 10% sucrose diet and transferred to new food every other day. During transfer, the number of dead flies is recorded to assess any differences in lifespan that may result from the exercise-training course. While the flies are being exercised, and while the climbing assay is being performed, flies are kept in a 25^0^ C room.

A minimum of 1200 flies should be collected and age-matched within + or – 1-2 days of each other. After collection, flies are divided so that 600 are in the experiment group and 600 are in the control group (20 flies per vial). Experimental flies are placed on the Power Tower and made to climb. Control flies are also placed on the machine, but with a sponge stopper pushed down into the vial approximately 1 cm above the flies to limit movement. This group serves as a control for effects the Power Tower treatment may have that are unrelated to exercise. When assaying outcomes across ages, it may also be important to add additional controls such as a 1) a group that is not placed on the machine at all and 2) a group that contains a sponge stopper pushed down without going on the machine.

Flies on the Power Tower are placed in vials containing 5 mL of food. This provides them with a softer landing. If flies are exercised in empty vials, they have a tendency to become sluggish and dazed, perhaps from the repeated stress of being knocked down. Sponge stoppers are used instead of cotton because the flies hang onto cotton and do not always drop down when the platforms drop.

Flies are exercised five days a week, utilizing a ramping schedule. During week one the flies are exercised for 2 hours/session, week two for 2.5 hours/session, and week three for three hours/session. Although other regimens have been tested, this protocol produces the most consistent results across genotypes. This protocol is, however, subject to a wide array of possible variations that might suit a particular experiment.

During and following training, flies are tested longitudinally in small cohorts (data should be kept for each vial separately) for changes in negative geotaxis ability (see below). In our standard protocol, flies are exercised for a total of three weeks and examined for a total of five weeks; three weeks during exercise-training and two weeks after cessation of exercise. We recommend charting negative geotaxis in all days of the timecourse, as this maximizes the possibilities for data analysis. Longitudinal experimental designs are easily facilitated by reserving the same group of flies for these tests throughout the timecourse. Optimally, additional tests that particular experimenters may wish to add should be done on sibling flies exercised at the same time.

Statistical Analysis

Negative geotaxis data for exercise and control flies was analyzed using the normalized index. For this method, individual averages for each vial over time are analyzed by 2-way ANOVA utilizing the JMP statistical software version 5. For cardiac stress resistance experiments at one time-point, a t-test assuming equal variances was used. To analyze rate of change over time, multivariate regression for treatment-by-age was employed.
